# *FOXP3* Contributes to TMZ Resistance, Prognosis, and Immune Infiltration in GBM from a Novel Pyroptosis-Associated Risk Signature

**DOI:** 10.1155/2022/4534080

**Published:** 2022-04-01

**Authors:** Lu Liang, Bin Yan, Yueying Liu, Shiyao Jiang, Hua He, Jingjing Huang, Wenbin Liu, Li Xie

**Affiliations:** ^1^Department of Head and Neck Surgery, Hunan Cancer Hospital, Xiangya School of Medicine, Central South University, Changsha, 410013 Hunan, China; ^2^School of Medicine, Hunan Normal University, Changsha, 410013 Hunan, China; ^3^Department of Pathology, The Second Clinical Medical College of Jinan University, The First Affiliated Hospital Southern University of Science and Technology, Shenzhen People's Hospital, 518020 Shenzhen, China

## Abstract

**Background:**

Pyroptosis is a form of programmed cell death, playing a significant role in cancer. Glioblastoma multiforme (GBM) is the most common malignant brain tumor. The poor prognosis in GBM due to temozolomide (TMZ) resistance has been widely discussed. Such being the case, the correlation between TMZ resistance and pyroptosis is seldom investigated. On this basis, this paper aims to explore the potential prognostic value of genes related to TMZ resistance and pyroptosis as well as their relationship to the immune microenvironment in GBM.

**Methods:**

A total of 103 patients from TCGA were assigned to a training cohort; 190 from CGGA were assigned to a validation cohort. The prognostic risk model reflecting pyroptosis and TMZ resistance was built from the training cohort using multivariate Cox regression and performed validation. RT-qPCR was used to examine the expression of 4 genes from the risk signature. *FOXP3* was selected for overexpression and verified using the western blot. The TMZ IC50 of *FOXP3*-overexpressed cell lines was determined by CCK8.

**Results:**

A four genes-based risk signature was established and validated, separating GBM patients into high- and low-risk groups. Compared with the low-risk group, the high-risk group presented worse clinical survival outcomes. Its differential expressed genes were enriched in immune-related pathways and closely related to the immune microenvironment. Moreover, RT-qPCR results suggested that *FOXP3*, *IRF3*, and *CD274* were significantly upregulated in TMZ-resistant strains, while *TP63* was downregulated. *FOXP3-*overexpressed GBM cell lines had higher TMZ IC50, implying an increased resistance of TMZ.

**Conclusion:**

A novel gene signature relevant to pyroptosis and TMZ resistance was constructed and could be used for the prognosis of GBM. The four genes from the risk model might play a potential role in antitumor immunity and serve as therapeutic targets for GBM.

## 1. Introduction

Glioblastoma (GBM), of the highest glioma grade, is one of the most common primary brain tumors (World Health Organization grade IV) [1], known for its highly malignancy and aggression and accounting for more than half of malignant glioma (MG) cases [2]. In addition, the prognosis for GBM is abysmal, mainly due to resistance to temozolomide (TMZ), resulting in the survival of less than a year of most patients, especially the elderly [3–5].

As a first-line chemotherapy agent for GBM patients after surgery, TMZ can increase the median overall survival of GBM patients from 12.1 to 14.6 months and improve the 2-year survival rate from 10.4% to 26.5% [6]. However, the prognosis of GBM remains exceedingly grim due to therapeutic resistance to TMZ [7]. Based on this, the identification of potential therapeutics to conquer TMZ resistance remains challenging in GBM [8, 9]. Encouragingly, evidence shows that pyroptosis is correlated with tumorigenesis and development and the resistance of chemotherapy drugs [10–12].

Pyroptosis is a form of programmed cell death distinguished from apoptosis by its proinflammatory nature [13, 14]. Pyroptosis has been proved to play a role in antitumor drug resistance in recent years. For instance, the treatment option for BRAF V600E/K-mutant melanoma is a combination of BRAF inhibitors and MEK inhibitors (BRAFi + MEKi). BRAFi + MEKi-resistant tumor lacks markers of pyroptosis and shows reduced T-cell infiltration within the tumor [15]. Another research reported that lobaplatin increases pyroptosis of cancer cells by inducing the degradation of cIAP1/2 during the treatment of nasopharyngeal carcinoma. The application of cIAP1/2 antagonists and lobaplatin may reduce chemotherapy resistance [16]. Thus, it is of great significance to explore the potential connection between pyroptosis and TMZ resistance in GBM.

Pyroptosis mediates the release of intracellular proinflammatory contents, which can produce and maintain a long-term chronic inflammatory tumor microenvironment (TME) [17, 18]. This internal environment also has a powerful influence on tumor progression and the antitumor immunity of the human body. Additionally, the inflammatory state of TME can influence the response of immune checkpoint therapy [18–20]. With the widespread use in clinical treatment and the positive feedback on outcomes, immune checkpoint blockade (ICB) has drawn increasing attention in GBM therapy [21–23]. Considering the currently limited treatment options and poor prognosis for GBM patients, further understanding of TME and ICB therapy becomes necessary [20].

This study identified differentially expressed genes (DEGs) in GBM relevant to pyroptosis and TMZ resistance using public databases like TCGA and CGGA. Then, a model for the high- and low-risk groups was constructed to predict GBM prognosis and investigate the link between the expression of relevant genes, including *FOXP3*, and immune infiltration. These will help to further deepen our knowledge of GBM and provide implications for individual immunotherapy of GBM.

## 2. Materials and Methods

### 2.1. Datasets Sources

**Figure 1:**
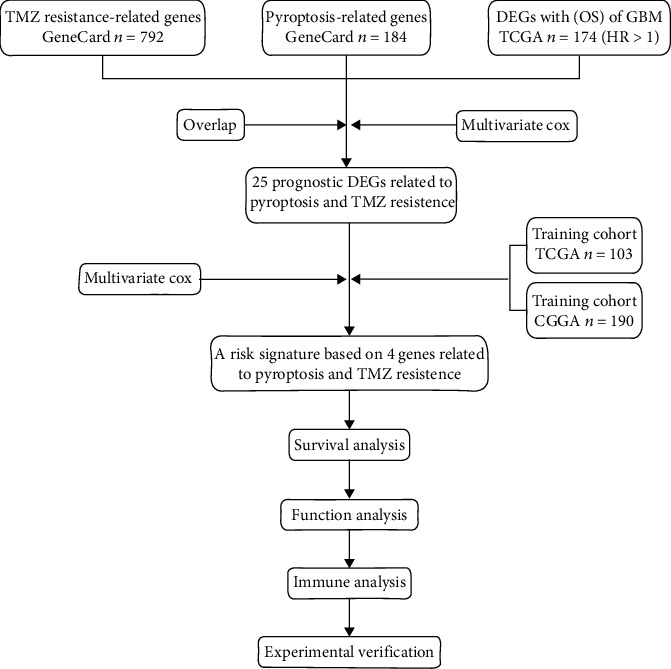
Workflow diagram.

Genes related to TMZ resistance or pyroptosis were derived from GeneCards (https://www.genecards.org/). DEGs with overall survival (OS) of GBM were derived from TCGA (https://portal.gdc.cancer.gov). TMZ-resistant samples from TCGA and CGGA were selected as the training cohort and the validation cohort, respectively. The normal samples were from GTEx (http://www.cgga.org.cn/). A statistical analysis of 293 clinical samples from the training and validation cohorts formed the following data: The number of patients aged 60 and above was 103, 35.15% of the total. Furthermore, the number of patients aged below 60 was 190, 64.85% of the total. The average ± standard deviation of the age for all patients was 51.96 ± 14.41. There were 111 female patients (37.88%) and 182 male patients (62.12%). Data analysis steps are shown in Figure 1.

### 2.2. Identification of Prognostic Model Genes

A total of 792 TMZ resistance-related genes and 184 pyroptosis-related genes were obtained from GeneCards. Among the 17621 DEGs in GBM patients with overall survival from TCGA, taking hazard rate (HR) higher than 1, 174 genes was obtained. Using the “Venn Diagram” R package, we identified 25 genes connected with TMZ resistance and pyroptosis in GBM. The R package “heat map” was utilized to compare the expression of the above genes in TMZ-resistant GBM samples and normal samples. The detailed data is provided in Supplementary Data 3. Then, multivariate Cox regression analysis was performed on 25 candidate genes relevant to pyroptosis and TMZ resistance. As a result, four optimal genes (*p* < 0.05) were screened for the prognostic model. The Kaplan-Meier (KM) curves were created separately to facilitate survival analysis with R package “survival.”

### 2.3. Construction and Validation of Gene Signature Model

A total of 103 TMZ-treated GBM samples from TCGA served as a training cohort, with each given an independent risk score. Based on multivariate Cox regression analysis and the selected gene expression levels, the following formula allowed us to calculate a risk score for each patient: risk score = expr_gene1_ × *β*_gene1_ + expr_gene2_ × *β*_gene2_ + ⋯+expr_gene N_ × *β*_gene N_. The median risk score was set as the split point, and the training cohort was then divided into high- and low-risk groups. The risk factor graph was generated by the R package “frisk.” Subsequently, the principal component analysis (PCA) was performed in the R package “stats” and visualized with “ggbiplot.” The OS between different groups was compared by KM analysis. Then, time-dependent ROC curve analysis was drawn by the R package “circlize” to verify the prognostic power of the risk signature. Finally, 190 TMZ-treated GBM samples from CGGA were taken as the validation cohort and validated using the same approach.

### 2.4. Assessment of the Independence of Risk Models

Multivariate Cox regression analysis determined whether risk score was an independent predictor of GBM prognosis. Forest plots for subgroup analysis were employed to identify the independence of the risk model. KM curves were produced using the R package “survival” to assess OS grouped by age and sex. The nomogram was plotted with the R package “survival” and “rms” to visualize the predictive model. Moreover, the calibrate curve was conducted with the R package “calibrate” to check the fit of the nomogram.

### 2.5. Analysis of the Infiltrating Immune Cells and Functional Enrichment

Based on the data acquired from TCGA, we used the ssGSEA algorithm to analyze the correlation between the expression levels of the 4 DEGs relevant to pyroptosis and TMZ resistance; we also performed immune cell-infiltrating analysis. Then, a volcano map was made using the “ggplot2” R package to identify DEGs in high- and low-risk groups and was applied for subsequent analysis. A functional analysis was carried out using the 388 protein-coding genes upregulated in the high-risk group (log FC>1). The Gene Ontology (GO) and Kyoto Encyclopedia of Genes and Genomes (KEGG) enrichment analysis were performed on differentially expressed protein-encoding upregulated genes in the high-risk group using the “clusterProfiler” R package. Moreover, the R package “circlize” was used to generate a chord diagram for KEGG. The data for GO enrichment analysis and KEGG pathway analysis are provided in Supplementary Data 4.

### 2.6. Immune Analysis

The Estimation of STromal and Immune cells in MAlignant Tumor tissues using Expression data (ESTIMATE) was used to calculate patients' ESTIMATE scores, immune scores, stromal scores, and tumor purity. MCPcounter was conducted to evaluate the abundance of 4 types of cells, including natural killer cells, neutrophils, myeloid dendritic cells, and fibroblasts. The CIBERSORT algorithm was employed to calculate the proportion of 22 kinds of tumor-infiltrating immune cells. Subsequently, expression levels of immune checkpoint molecules in the high- and low-risk groups were visualized as boxplots utilized by the “ggpubr” R package.

### 2.7. Cell Lines, Cell Culture, and Plasmids

Glioma cell lines LN229 (ATCC: CRL-2611™) and U87MG (ATCC: HTB-14™) were derived from the American Type Culture Collection (ATCC). All cell lines were cultured in Dulbecco's Modified Eagle's Medium (DMEM, Biological Industries, Israel), supplemented with 10% fetal bovine serum (FBS, Biological Industries, Israel), and maintained at 37°C with 5% CO_2_. All cell lines were tested negative for mycoplasma contamination and were passaged less than 10 times after the initial recovery of the frozen stocks. All cell lines were validated by short tandem repeat profiling before use.

We used the TMZ dose-escalation method to acquire drug-resistant cell lines. The parental cell lines, i.e., LN229 and U87MG, were initially exposed to 10 *μ*M TMZ for one week [24]. Depending on their growth status, the cells then underwent repeated exposure to progressively increasing amounts of TMZ (5-20 *μ*M) [25] until 5-fold 50% inhibitory concentration (IC50) was reached [26]. The established TMZ-resistant cell lines were named LN229-R and U87MG-R. The above process took six months [27, 28].

*FOXP3* lentiviral construct was generated by inserting the *FOXP3* cDNA into the pLVX-EF1alpha-IRES-Puro vector (catalog no. 631988; Clontech, Mountain View, CA) using restriction enzymes EcoRI and BamHI (Takara). The short-hairpin RNA (shRNA) vector was plvx-shRNA1, and the targeted sequences of shRNA for *FOXP3* were as follows: contol (CACTTACGCTGAGTACTTCGA), sh*FOXP3*#1 (AGCTGGAGTTCCGCAAGAAAC), and sh*FOXP3*#2 (TCCTACCCACTGCTGGCAAAT).

### 2.8. Quantitative Real-Time PCR (RT-qPCR)

A total of 1 ml Trizol reagent (R401-01, Vazyme, Nanjing, China) was added to the target cells for lysis to obtain RNA. The HiScript® II Q RT SuperMix for qPCR (+gDNA wiper) (R223-01, Vazyme, Nanjing, China) was used to synthesize the first-strand cDNA. Quantitative PCR was performed using the real-time PCR System (CFX Connect, Bio-Rad, USA) with MonAmp™ ChemoHS qPCR Mix (MQ00401S, Monad, Shanghai, China). The primers for this experiment are derived from Sangon (Shanghai, China), and primer sequences are presented in Table 1. The presented results were subjected to at least 3 experiments.

### 2.9. Western Blot

The proteins of the target cells were extracted using the RIPA lysis buffer (Beyotime, Guangzhou, China), of which 40 *μ*g was taken for western blot analysis. Rabbit monoclonal antibody antihuman *FOXP3* (#12632, CST), mouse monoclonal antibody antihuman *β*-actin (#AF7018, Affinity), goat antirabbit antibody (#S0001, Affinity), and goat antimouse antibody (#A21010, Abbkine) were purchased.

### 2.10. Cell Viability Assay

Cells to be tested were seeded in 96-well plates at a density of 2000 cells/well. The *FOXP3* overexpression cell lines were incubated for 24 hours, and the cells were treated with TMZ with a concentration gradient of 1 *μ*M, 5 *μ*M, 25 *μ*M, 75 *μ*M, 100 *μ*M, 200 *μ*M, 1000 *μ*M, and 2000 *μ*M and cultured for 48 hours. Cell Counting Kit-8 (CCK-8, A311-01, Vazyme, Nanjing, China) was added to the cells (10 *μ*l/well), which were incubated for 2 hours. The absorbance at 450 nm was measured using a microplate reader (Synergy2, Bio-Tek, USA). The TMZ-resistant strains knocked down by *FOXP3* were incubated for 2 hours at 0 h, 24 h, 48 h, and 72 h with CCK-8 (10 *μ*l/well). Then, proliferation was detected using a microplate reader. Finally, we obtained the IC50 value of *FOXP3* overexpression cell lines, and the proliferation curve of *FOXP3* knockdown TMZ-resistant strains was plotted using the GraphPad Prism (version 9.1.0.221).

### 2.11. Statistical Analysis

Data were presented as mean ± standard deviation. The Student *t*-test was used to analyze differences between groups. Multivariate Cox regression was used to determine independent prognostic factors for OS. Additionally, the log-rank test was applied to compare the survival of the groups. Data analyses and visualization were mainly completed using R (version 4.1.2), with *p* < 0.05 considered statistically significant.

## 3. Results

### 3.1. Identification of DEGs Relevant to Pyroptosis and TMZ Resistance

**Figure 2:**
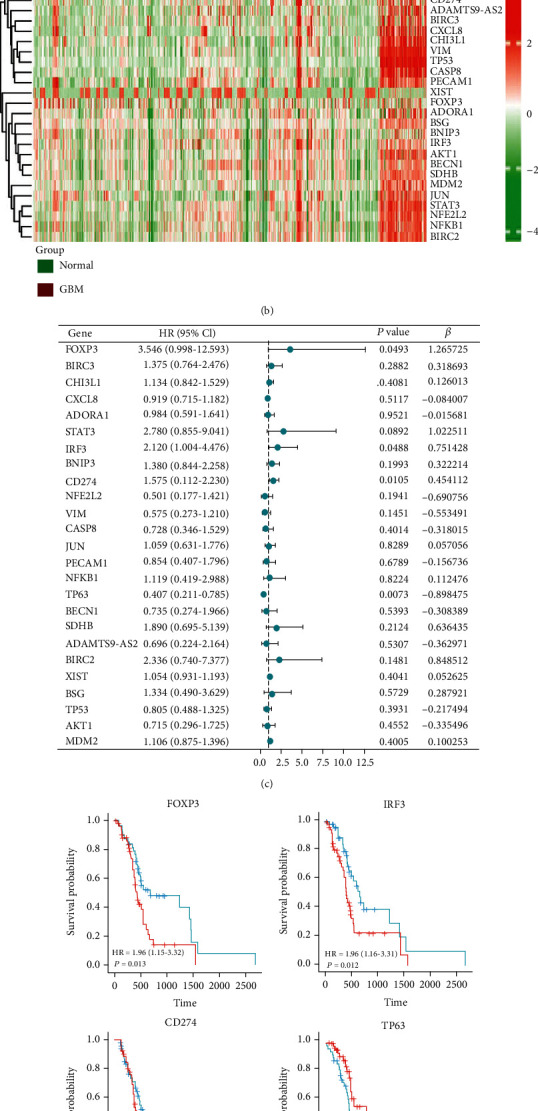
Identification of DEGs related to prognostic pyroptosis and TMZ resistance in glioblastoma. (a) Venn diagram to identify the TMZ resistance 79 (GeneCards), pyroptosis 184 (GeneCards), and DEGs 17621. (b) Twenty-five overlapping genes showed high expression in TMZ-treated GBM samples compared to normal samples. (c) Forest plots presented the multivariate Cox regression analysis results between 25 overlapping genes and OS. (d) KM curves analyzed the prognosis of samples expressing 4 genes related to pyroptosis and TMZ resistance.

Genes related to pyroptosis (Supplementary Data 1) or TMZ resistance (Supplementary Data 2) were obtained from GeneCards. GBM DEGs for OS were obtained from analysis of TCGA (HR>1). Here, we found 25 genes (*FOXP3*, *BIRC3*, *CHI3L1*, *CXCL8*, *ADORA1*, *STAT3*, *IRF3*, *BNIP3*, *CD274*, *NFE2L2*, *VIM*, *CASP8, JUN*, *PECAM1*, *NFKB1*, *TP63*, *BECN1*, *SDHB*, *ADAMTS9AS2*, *BIRC2*, *XIST*, *BSG*, *TP53*, *AKT1*, and *MDM2*) in all three sets (Figure 2(a)). We downloaded 766 normal tissue samples from the GTEx database and 103 TMZ-treated GBM samples from the TCGA database. The comparison in a heat map reveals that the 25 candidate genes in the normal group were expressed at a lower level than those in the TMZ-treated tumor samples (Figure 2(b)). To construct a high-quality prognostic risk signature, we used multivariate Cox regression analysis to identify 4 DEGs significantly related to pyroptosis and TMZ resistance (*FOXP3*, *IRF3*, *CD274*, and *TP63*) (Figure 2(c), *p* < 0.05). The KM curves of *FOXP3*, *IRF3* low expression group, and *TP63* high expression group indicated a superior prognosis (*p* < 0.05), while that of *CD274* was not statistically significant (Figure 2(d)).

### 3.2. Development of a Prognostic Risk Signature Based on DEGs

**Figure 3:**
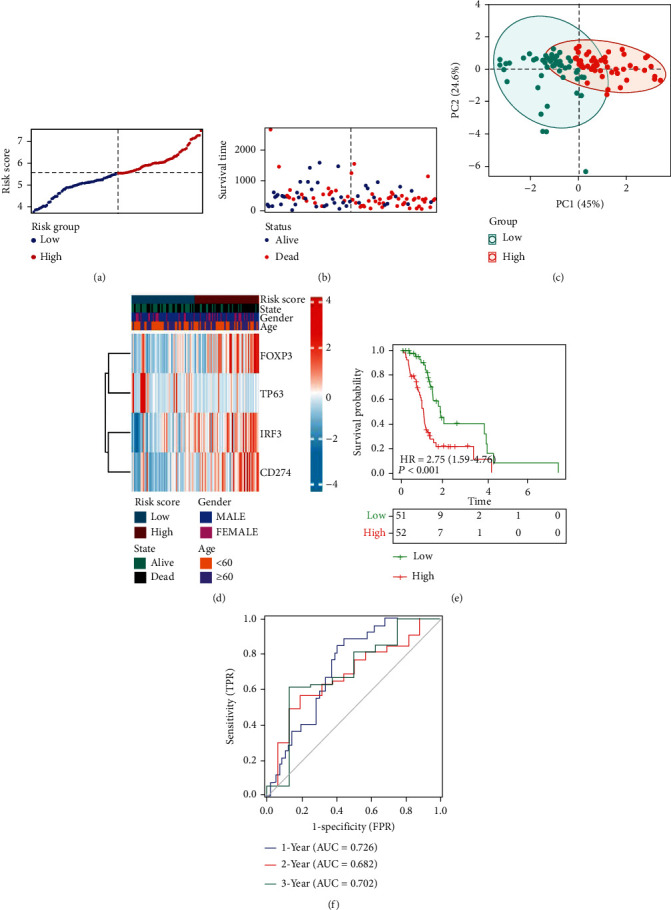
Training cohorts to develop predictive DEG-based risk models. (a) The distribution and split point of the risk scores in the training cohort. (b) The OS of the risk scores in the training cohort. (c) PCA plot of the training cohort proved the feasibility of grouping. (d) The heat map of differences in the expression of 4 genes in the high- and low-risk groups of training cohorts. (e) In the training cohort, KM curves showed a better prognosis of patients in the low-risk group. (f) The area under the time-dependent ROC curves validated the accuracy of the risk score in predicting prognosis in the training cohort.

Based on the above 4 genes, the relevant samples from the TCGA database were divided into high-risk and low-risk groups according to median risk scores (Figure 3(a)). We found that patients in the high-risk group had a higher mortality rate than those in the low-risk group (Figure 3(b)). Moreover, the PCA plot showed significant differences of both the two groups (Figure 3(c)), proving the validity of the grouping. A heat map was used to visualize the gene expression profile from the risk model. It could be seen that patients in the high-risk group tended to express risk genes with high-risk scores, including *FOXP3*, *IRF3*, and *CD274*. In contrast, patients in the low-risk group expressed protective genes with low-risk scores, including *TP63* (Figure 3(d)). KM curves suggested that patients in the high-risk group had a lower survival rate (Figure 3(e), *p* < 0.05). Regarding the estimation of risk prediction models using the time-dependent ROC curves, the areas under the curve of ROC (AUC) reached 0.726 at one year, 0.682 at two years, and 0.702 at 3 years (Figure 3(f)). The above results indicated that the gene signature model associated with pyroptosis and TMZ resistance could realize an accurate prognosis of GBM.

**Figure 4:**
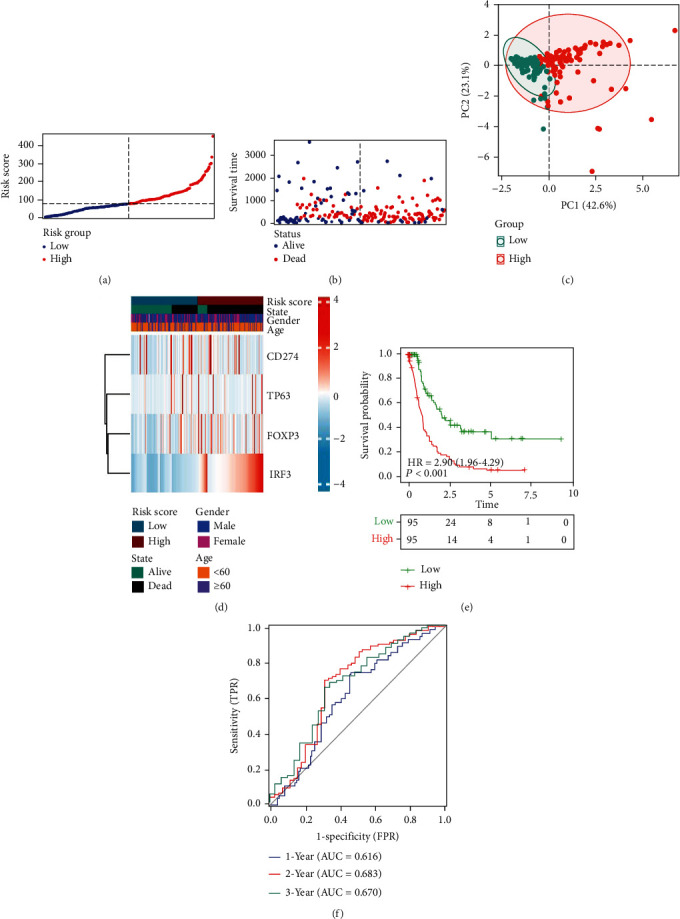
Validation cohort to develop DEG-based predictive risk models. (a) The distribution and split point of the risk scores in the validation cohort. (b) The OS of the risk scores in the validation cohort. (c) PCA plot of the validation cohort proved the feasibility of grouping. (d) The heat map of differences in the expression of 4 genes in the high- and low-risk groups of validation cohorts. (e) In the validation cohort, KM curves showed that patients in the low-risk group had a better prognosis. (f) The area under the time-dependent ROC curves validated the accuracy of the risk score in predicting prognosis in the training cohort.

The grouping of the validation cohort was similar to that of the training cohort (Figures 4(a)–4(c)). The heat map displayed that expression of *FOXP3* and *IRF3* was higher in the high-risk group, while the expression of *CD274* and *TP63* showed no significant difference in the high- and low-risk groups (Figure 4(d)). The KM survival curve showed that similar to the training cohort, the low-risk group presented a significantly higher survival probability than the high-risk group (Figure 3(e), *p* < 0.05). The AUC was 0.616, 0.683, and 0.670 at 1, 2, and 3 years, respectively (Figure 4(f)).

### 3.3. Independent Prognostic Value of the Risk Model

**Figure 5:**
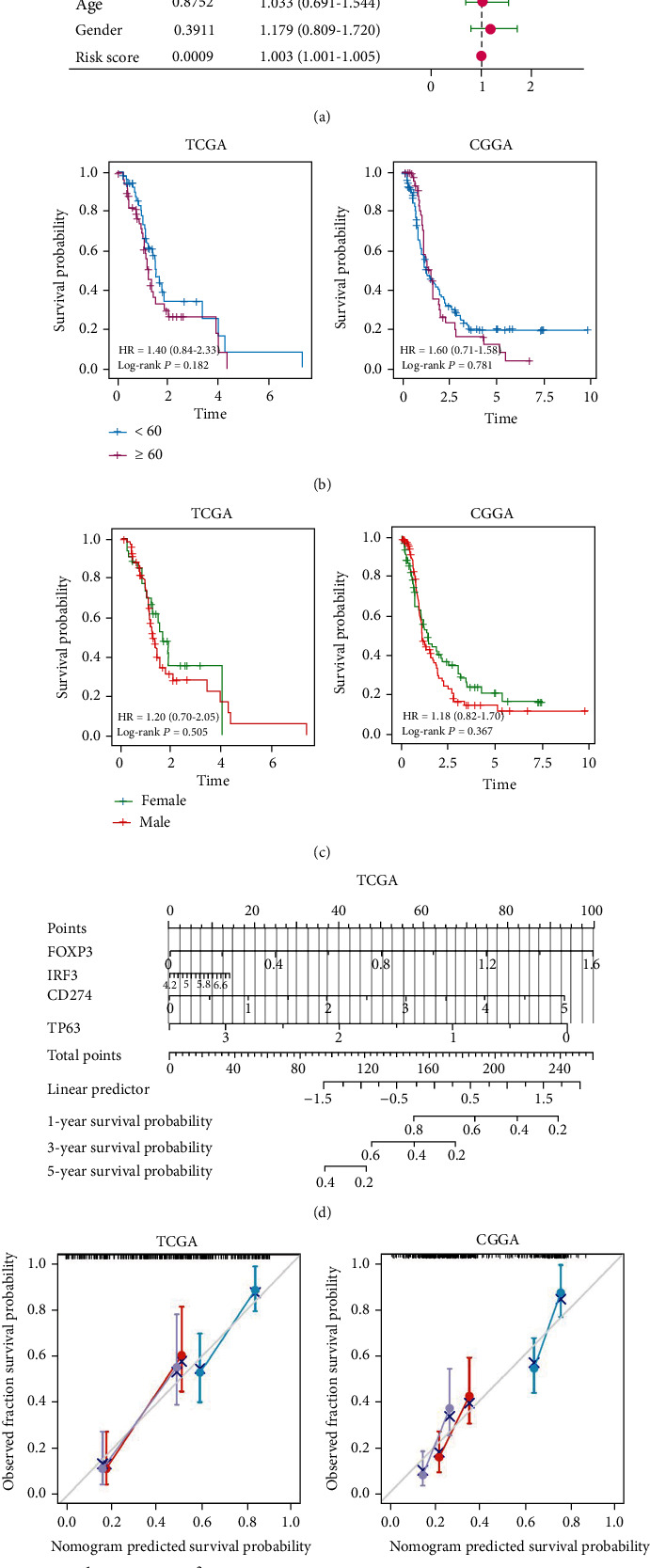
Risk model as an independent indicator of GBM prognosis. (a) Multivariate Cox regression results showed that risk score was an independent factor in GBM prognosis for patients in the training and validation cohorts. (b) KM curves with age as a subgroup were not statistically significant. (c) KM curves with gender as a subgroup were not statistically significant. (d) Nomogram based on the genes related to pyroptosis and TMZ resistance quantitatively predicted the survival probability of GBM patients. (e) Calibration curves were plotted separately using data from TCGA and CGGA databases to verify the accuracy of the nomogram.

To further clarify whether risk score was an independent factor in the GBM prognosis, we used multivariate Cox regression to analyze patients' clinical characteristics and risk scores. The results showed that the risk score was statistically significant for GBM survival in the validation and training cohort (training cohort: HR = 2.066, 95% CI = 1.500-2.845, *p* < 0.05; validation cohort: HR = 1.003, 95% CI = 1.001-1.005, *p* < 0.05; Figure 5(a)). In addition, KM curve analysis was conducted on subgroups of samples with different clinical characteristics. However, as shown in Figures 5(b) and 5(c), the KM curves grouped by age and gender in the training and validation cohorts were not statistically significant. To quantitatively predict the survival rate of GBM patients, we constructed a prognostic nomogram based on the 4 genes in the risk model (Figure 5(d)). The total score was obtained by summing the scores of each prognostic gene in the nomogram, which in turn could be used to calculate 1-, 3-, and 5-year survival rates of GBM patients. In addition, the calibration curve also indicated the accuracy of the nomination diagram (Figure 5(e)). Eventually, we concluded that the risk score was an independent factor in the GBM prognosis and the risk signature had independent predictive value.

### 3.4. Infiltrating Immune Cells and Functional Enrichment Based on the Risk Model

**Figure 6:**
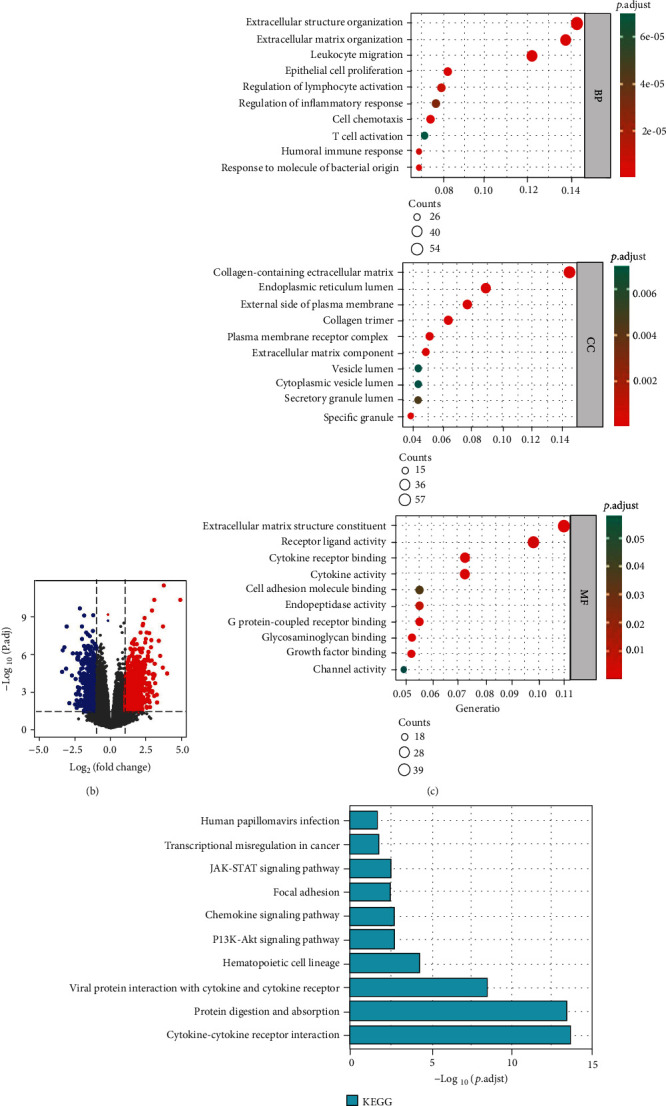
Functional enrichment analysis. (a) Correlation of each of the 4 genes related to pyroptosis and TMZ resistance with immune infiltrating cells. (b) Volcano plot depicting DEGs in the high- and low-risk groups in the training cohort. (c) GO analysis suggested a considerable enrichment of DEGs in immune-related biological processes (BP). (d) KEGG analysis indicated that DEGs were enriched in immune-related pathways. (e) Chord diagram of KEGG. ∗*p* < 0.05; ∗∗*p* < 0.01; ∗∗∗*p* < 0.001; ns *p* > 0.05.

The correlation between genes related to pyroptosis and TMZ resistance and infiltrating immune cells was further explored. Obviously, 4 genes were closely associated with infiltrating immune cells (Figure 6(a)). For instance, *FOXP3* was linked to regulatory T cells, dendritic cells, and T cells. On this basis, it is necessary to conduct further research on immune.

To research the molecular mechanisms of genes related to pyroptosis and TMZ resistance, GO and KEGG analysis was performed based on our risk model. Initially, differential analysis of the TCGA data between the high- and low-risk groups identified 1077 upregulated genes and 590 downregulated genes (Figure 6(b)). Subsequently, 388 protein-coding genes upregulated in the high-risk group were selected for functional analysis. GO analysis suggested that DEGs were mainly enriched in immune-related biological processes (BP), such as leukocyte migration, regulation of lymphocyte activation, T cell activation, and humoral immune response. (Figure 6(c)). The KEGG pathway analysis indicated that DEGs were enriched in the JAK-STAT signaling pathway, chemokine signaling pathway, and PI3K-Akt signaling pathway (these are associated with the immune pathway) (Figures 6(d) and 6(e)). Taken together, the high-risk score group was strongly associated with immunoregulatory.

### 3.5. Comparison of the Immune Activity between High- and Low-Risk Group

**Figure 7:**
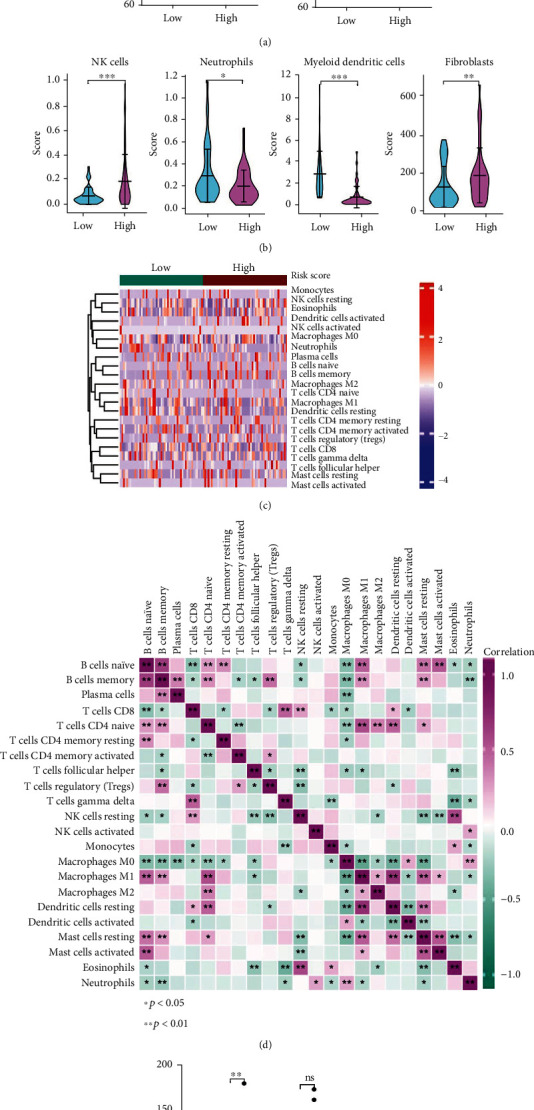
Immune analysis for high- and low-risk groups based on training cohort. (a) Estimation of STromal and Immune cells in MAlignant Tumor tissues (ESTIMATE) algorithm. (b) The violin plot revealed the abundance of the 4 types of immune cells. (c)–(d) Heat map of the situation and correlation of tumor-infiltrating immune cells. (e) The expression levels of immune checkpoints in high- and low-risk groups. ∗*p* < 0.05; ∗∗*p* < 0.01; ∗∗∗*p* < 0.001; ns *p* > 0.05.

The ESTIMATE method was used to assess the association between the risk signature and the immune microenvironment of GBM. As shown in Figure 7(a), the low-risk group represents a higher ESTIMATE, immune score, and stromal score and lower tumor purity than the high-risk group (*p* < 0.05). Regarding the abundance of 4 types of immune cells, MCPcounter revealed that NK cells (*p* < 0.001) and fibroblasts (*p* < 0.01) were higher in the high-risk group; in contrast, neutrophils (*p* < 0.05) and myeloid dendritic cells (*p* < 0.001) were higher in the low-risk group (Figure 7(b)). The CIBERSORT algorithm was used to evaluate the status of the 22 tumor-infiltrating immune cells. A significant difference could be observed in partial immune cell infiltration between the two groups, such as dendritic cell activation. Additionally, naive and memory B cells, activated mast cells and naive B cells, and naive CD4 T cells and M1 macrophages represented significant correlation (Figures 7(c) and 7(d)). Interestingly, the *FOXP3* gene was associated with activation of various naive cells [29]. Then, we compared the molecular expression levels of joint and immune checkpoints in the high- and low-risk groups. As illustrated in Figure 7(e), the immune checkpoint molecule expression levels are higher in the high-risk group than in the low-risk group, such as PDL1 (*p* < 0.001), CTLA4 (*p* < 0.01), and LAG3 (*p* <0.05). The above findings demonstrated that genes related to the expression of pyroptosis and TMZ resistance were associated with the immune environment of GBM patients. Furthermore, those genes also acted on the immune checkpoint. This finding may provide a new idea to ICB therapy for GBM patients.

### 3.6. Experimental Verification Revealing *FOXP3* Involved in TMZ Resistance

**Figure 8:**
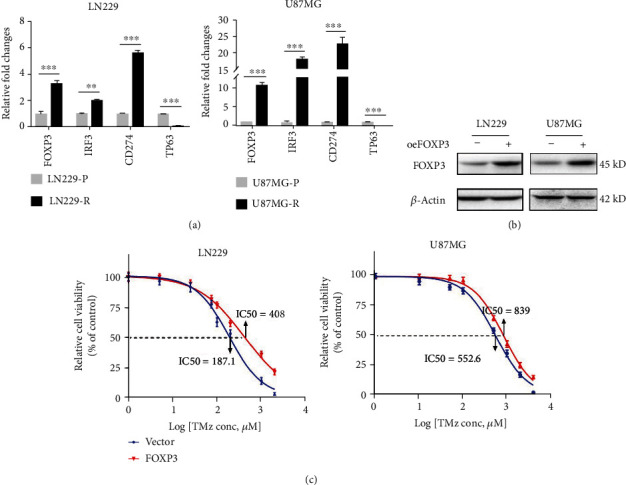
Experimental verification reveals *FOXP3* involved in TMZ resistance (a). RT-qPCR analysis displayed the expression level of 4 genes in GBM-resistant strains and normal groups. (b) *FOXP3* stable overexpression by LN229 and U87MG cells was verified by western blot. (c). The cell viability assay revealed that *FOXP3-*overexpressed GBM cell lines had a higher IC50 than the control group. ∗*p* < 0.05; ∗∗*p* < 0.01;∗∗∗*p* < 0.001; ns *p* > 0.05.

To verify the expression of the 4 genes in GBM-resistant strains, we performed further experiments. RT-qPCR results showed high expression of *FOXP3*, *IRF3*, and *CD274* and low expression of *TP63* in resistant strains, i.e., LN229 (LN229-R) and U87MG (U87MG-R) (Figure 8(a)), which coincided with the expression levels of the 4 genes depicted in the training cohort's high-risk and low-risk groups (Figure 3(d)). Taken together, in GBM-resistant strains, *FOXP3*, *IRF3*, and *CD274* tended to be highly expressed, while *TP63* tended to be low expressed. Subsequently, *FOXP3*-overexpressing LN229 and U87MG constructed by lentiviral infection were verified by western blot (Figure 8(b)). Cell viability assay suggested that the IC50 value of *FOXP3*-overexpressing LN229 was 408 *μ*M, 220.9 more than the 187.1 *μ*M of the vector group. In contrast, the IC50 of *FOXP3-*overexpressing U87MG was 839 *μ*M, 286.4 more than the 552.6 *μ*M of vector control (Figure 8(c)). This indicated that LN229 and U87MG overexpressed *FOXP3*, increasing IC50 for TMZ and drug resistance but reducing drug sensitivity. Moreover, stable knockdown *FOXP3* cell lines were constructed from LN229-R and U87MG-R using the shRNA strategy. RT-qPCR was performed to detect the knockdown efficacy, and both sh*FOXP3*#1 and #2 were found to inhibit *FOXP3* expression significantly. (Supplementary Figure S1 A). Then, CCK-8 assay was carried out using stable knockdown *FOXP3* cell lines, indicating that knockdown of *FOXP3* markedly inhibited cell proliferation (Supplementary Figure S1 B). Based on the above experimental conclusions, 4 genes related to pyroptosis and TMZ resistance were expressed at high or low levels in glioma-resistant strains, affecting the therapeutic efficacy of TMZ on samples. Perhaps these 4 genes will become an essential point to solve TMZ resistance and improve the prognosis in GBM patients.

## 4. Discussion

This study screened out DEGs related to pyroptosis and TMZ resistance in GBM. By analyzing the relations between the expression of the 4 DEGs and OS of GBM patients, a novel signature model was constructed. The signature was built by dividing the training cohort of the TCGA database sample into high- and low-risk groups and was validated in the validation cohort. Then, we performed differential analysis between the two groups. Enrichment analysis revealed that the protein-encoding upregulated genes in the high-risk group were concentrated in immune-related pathways.

Pyroptosis-mediated inflammatory response elicits robust antitumor immunity in the microenvironment and acts synergistically with ICB, such as PD1 [30, 31]. PD-L1 can mediate pyroptosis through non-immune checkpoint function, leading to tumor necrosis [32]. TME, which consists of non-malignant cells (such as endothelial, immune, and inflammatory cells), may mediate chemo- and radio-therapeutic resistance through multiple mechanisms [33, 34]. Cellular crosstalk and cell-to-TME-matrix interaction lead to acquired multi-drug resistance. For example, endothelial cells can selectively upregulate T cell inhibitory receptors and participate in immune evasion in GBM [35, 36]. Additionally, the mechanism of de novo resistance is that the stromal tissue within the TME mediates immune evasion of tumor cell subsets and enables them to resist to chemotherapy by inducing stemness [37–39]. TMZ, an oral alkylating agent, has been widely used in postoperative chemotherapy for GBM. Overexpression of O6-methylguanine-DNA methyltransferase (MGMT) or mismatch repair (MMR) deficiency leads to rapid acquired TMZ resistance for GBM, which is a significant contributor to tumor recurrence [27, 40, 41]. Improvement of the sensitivity of GBM patients to TMZ has been extensively researched. However, little research was found on the relationship between pyroptosis and TMZ resistance in GBM. On this basis, we attempted to discuss the above two hot topics in the context of GBM TME. This study constructed prognostic risk models using the screened 4 genes related to pyroptosis and TMZ resistance (i.e., *FOXP3*, *IRF3*, *CD274*, and *TP63*).

Related research suggests that *FOXP3* exerts a paradoxical effect on tumorigenesis. For one thing, *FOXP3* is a tumor suppressor of breast cancer and prostate cancer [29, 42, 43]. For another, the expression of *FOXP3* correlates with poor prognosis. For example, high-level *FOXP3* contributes to the proliferation and metastasis of non-small cell lung cancer cells [44]. Additionally, Chun Li et al. found that the downregulation of *FOXP3* in human lung adenocarcinoma inhibited cell proliferation and enhanced chemosensitivity [45]. The transcription factor *IRF3* is essential for innate antiviral immunity. *IRF3* is a critical yes-associated protein (YAP) activator, probably involved in GBM chemoresistance via the Hippo pathway [46, 47]. CD274, the gene encoding PD-L1, is commonly used in immunotherapy and presents effectiveness against many cancer types [48, 49]. As previously mentioned, CD274 is involved in the antitumor immunity of GBM. The inhibition of MGMT responsible for the mediation of p53 activation is found to have a strong association with the inhibition of glioblastoma resistance to TMZ [50, 51]. TP63 is a member of the P53 family, whose expression can affect the expression of TP53 [52]. TAp63, the isoform of TP63, has been shown to facilitate TMZ sensitivity in GBM cells through down-regulation of MYC [53]. Taken together, the available research indicated that *FOXP3*, *IRF3*, *CD274*, and *TP63* play an essential role in cancer, even in GBM.

The prognostic signature constructed for the 4 genes related to pyroptosis and TMZ resistance presented some predictive power and good model fit (0.616, 0.683, and 0.670 at 1, 2, and 3 years, respectively). Further analysis found that the risk score was an independent predictor of OS in GBM patients. These findings will contribute to providing an effective prognostic prediction for GBM patients. Then, the expression of *FOXP3*, *IRF3*, *CD274*, and *TP63* was validated by RT-qPCR and western blotting. Furthermore, cell viability assay revealed that *FOXP3* was involved in TMZ resistance in GBM.

The development of a new signature has advanced GMB research. However, we still have to acknowledge the limitations of this study. For example, differences between the sample sources of TCGA and CGGA databases led to a little inconsistent expression of CD274 and TP63 in training and validation cohorts. This research only initially revealed the correlation between DEGs and antitumor immunity, and further understanding of molecular mechanisms needs to be refined by more experiments in the future. Moreover, the tumor immune environment and ICB therapy deserve more in-depth study.

## 5. Conclusion

To sum up, a novel prognostic signature based on 4 genes related to pyroptosis and TMZ resistance and correlated with GBM OS could be applied to predict GBM prognosis. In addition, the immune analysis of GBM patients suggested that *FOXP3* was involved in TMZ resistance of GBM, which was verified experimentally. The analysis result is expected to provide some insights into the immunotherapy of patients.

## Figures and Tables

**Table 1:** Primers for qPCR.

Primers	Forward	Reverse
*FOXP3*	5′-GGCACAATGTCTCCTCCAGAGA-3′	5′-CAGATGAAGCCTTGGTCAGTGC-3′
*IRF3*	5′-TCTGCCCTCAACCGCAAAGAAG-3′	5′-TACTGCCTCCACCATTGGTGTC-3′
*CD274*	5′-TGCCGACTACAAGCGAATTACTG-3′	5′-CTGCTTGTCCAGATGACTTCGG-3′
*TP63*	5′-CAGGAAGACAGAGTGTGCTGGT-3′	5′-AATTGGACGGCGGTTCATCCCT-3′
*β-actin*	5′-CACCATTGGCAATGAGCGGTTC-3′	5′-AGGTCTTTGCGGATGTCCACGT -3′

## Data Availability

The datasets supporting the conclusions of this article are available in the GeneCards (https://www.genecards.org/), TCGA database (https://portal.gdc.cancer.gov), CGGA database (http://www.cgga.org.cn/), and GTEx (https://www.genome.gov/).

## Abbreviations

GBM: Glioblastoma multiforme
TMZ: Temozolomide
MGs: Malignant gliomas
TME: Tumor microenvironment
ICB: Immune checkpoint blockade
OS: Overall survival
DEGs: Differentially expressed genes
KM: Kaplan-Meier
PCA: Principal component analysis
GO: Gene Ontology
KEGG: Kyoto Encyclopedia of Genes and Genomes
IC50: 50% inhibitory concentration
BP: Biological process
MGMT: O6-methylguanine-DNA methyltransferase
MMR: Mismatch repair
YAP: Yes-associated protein.

## Supplementary Materials

Supplementary Materials Supplementary Data 1: TMZ resistant-related genes derived from GeneCards. Supplementary Data 2: Pyroptosis-related genes derived from GeneCards. Supplementary Data 3: Original data for the Figure 1(b) heat map. Supplementary Data 4: Original data for the Figure 5(c) GO and KEGG analysis. Supplementary Figure S1: Experimental verification revealed that *FOXP3* was involved in TMZ resistance
