# Supplementary material for: FOXP3 Contributes to TMZ Resistance, Prognosis, and Immune Infiltration in GBM from a Novel Pyroptosis-Associated Risk Signature
Source: Dis Markers. 2022 Apr 1;2022:4534080. doi: 10.1155/2022/4534080 (PMC8993549; doi:10.1155/2022/4534080)
Supplement: Supplementary Materials — Supplementary Data 1: TMZ resistant-related genes derived from GeneCards. Supplementary Data 2: Pyroptosis-related genes derived from GeneCards. Supplementary Data 3: Original data for the Figure 1(b) heat map. Supplementary Data 4: Original data for the Figure 5(c) GO and KEGG analysis. Supplementary Figure S1: Experimental verification revealed that FOXP3 was involved in TMZ resistance [file 4534080.f1.zip › Supplementary Figure S1.docx]

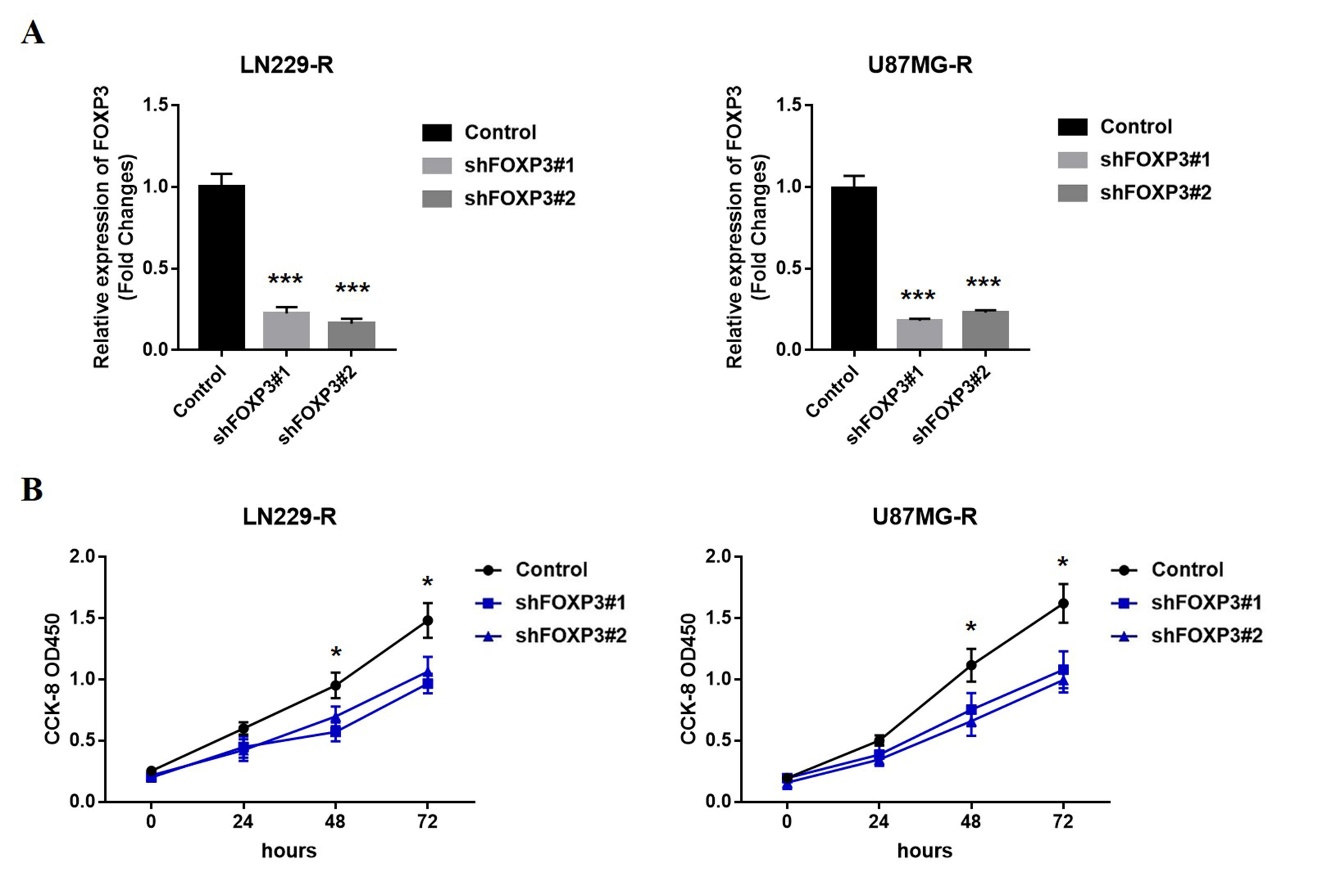


**Supplementary Figure S1** Experimental verification revealed that *FOXP3* was involved in TMZ resistance

(A) *FOXP3* stable knockdown by LN229-R and U87MG-R cells was verified by RT-qPCR. (B) The CCK-8 assay indicated that knockdown of *FOXP3* markedly inhibited cell proliferation. **p* < 0.05; ** *p* < 0.01; *** *p* < 0.001; ns *p* > 0.05.
