# Supplementary figures and images for: FOXP3 Contributes to TMZ Resistance, Prognosis, and Immune Infiltration in GBM from a Novel Pyroptosis-Associated Risk Signature
Source: Dis Markers. 2022 Apr 1;2022:4534080. doi: 10.1155/2022/4534080 (PMC8993549; doi:10.1155/2022/4534080)

**A**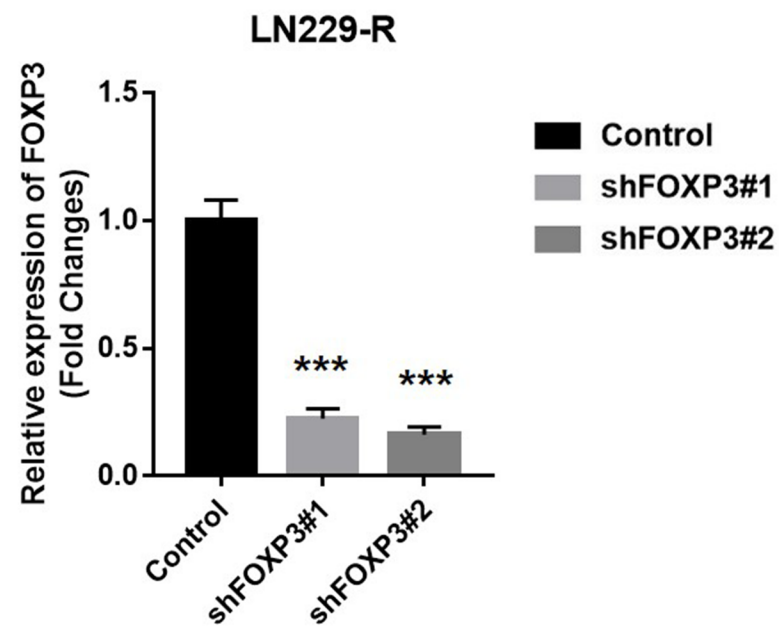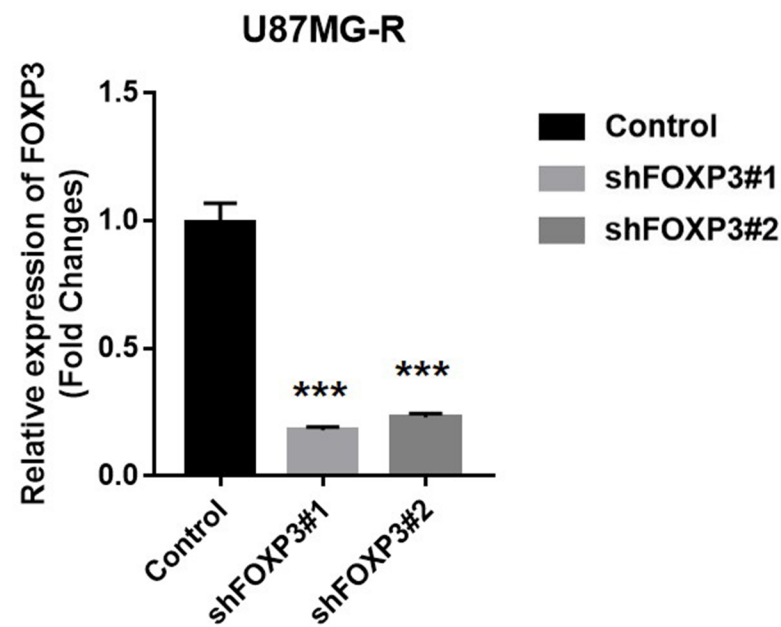**B**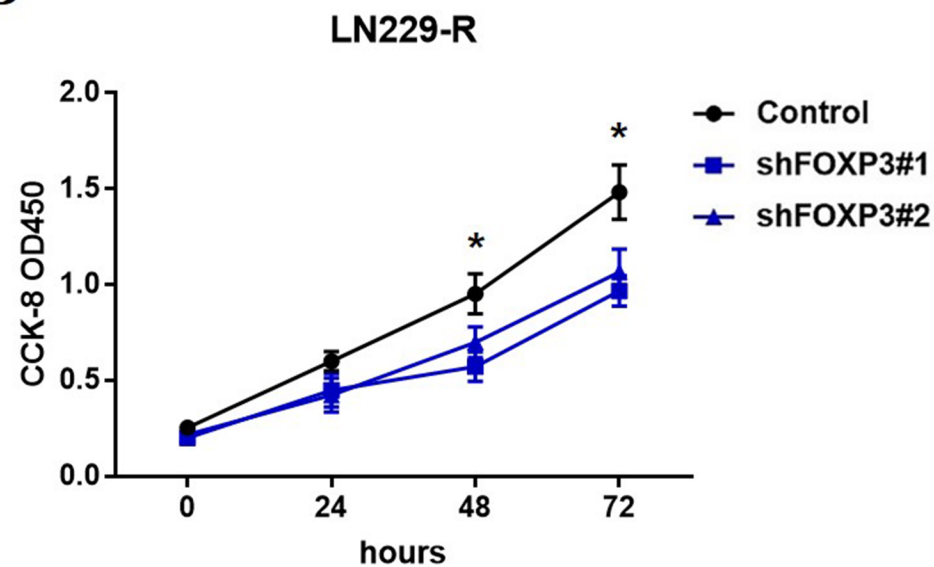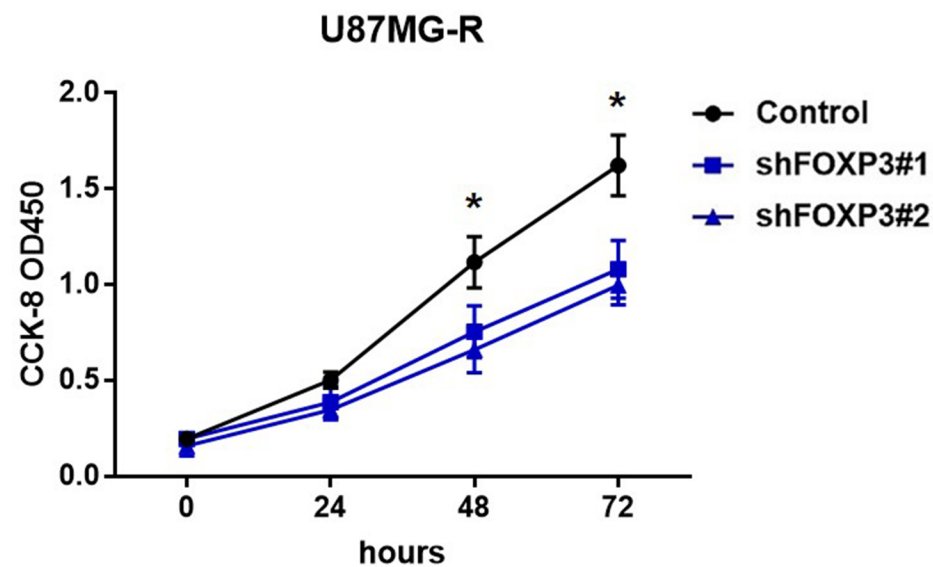

Supplement: Supplementary Materials — Supplementary Data 1: TMZ resistant-related genes derived from GeneCards. Supplementary Data 2: Pyroptosis-related genes derived from GeneCards. Supplementary Data 3: Original data for the Figure 1(b) heat map. Supplementary Data 4: Original data for the Figure 5(c) GO and KEGG analysis. Supplementary Figure S1: Experimental verification revealed that FOXP3 was involved in TMZ resistance [file 4534080.f1.zip › Figure S1 (1).pdf]
